# Understanding neutralising antibodies against SARS-CoV-2 and their implications in clinical practice

**DOI:** 10.1186/s40779-021-00342-3

**Published:** 2021-08-31

**Authors:** Natalie Yan-Lin Pang, Alexander Shao-Rong Pang, Vincent T. Chow, De-Yun Wang

**Affiliations:** 1 Yong Loo Lin School of Medicine, National University of Singapore, Singapore, 119228 Singapore; 2 Department of Microbiology and Immunology, National University of Singapore, Science Drive 2, Singapore, 117545 Singapore; 3 Infectious Diseases Translational Research Program, Yong Loo Lin School of Medicine, National University of Singapore, Singapore, 119228 Singapore; 4 Department of Otolaryngology, Yong Loo Lin School of Medicine, National University of Singapore, 1E Kent Ridge Road, Singapore, 119228 Singapore

**Keywords:** SARS-CoV-2, Coronavirus disease 2019, Neutralising antibodies, Persistence, Spike glycoprotein, Receptor-binding domain, B cells, T cells, Convalescent plasma

## Abstract

SARS-CoV-2 is a newly identified member of the coronavirus family that has caused the Coronavirus disease 2019 (COVID-19) pandemic. This rapidly evolving and unrelenting SARS-CoV-2 has disrupted the lives and livelihoods of millions worldwide. As of 23 August 2021, a total of 211,373,303 COVID-19 cases have been confirmed globally with a death toll of 4,424,341. A strong understanding of the infection pathway of SARS-CoV-2, and how our immune system responds to the virus is highly pertinent for guiding the development and improvement of effective treatments. In this review, we discuss the current understanding of neutralising antibodies (NAbs) and their implications in clinical practice. The aspects include the pathophysiology of the immune response, particularly humoral adaptive immunity and the roles of NAbs from B cells in infection clearance. We summarise the onset and persistence of IgA, IgM and IgG antibodies, and we explore their roles in neutralising SARS-CoV-2, their persistence in convalescent individuals, and in reinfection. Furthermore, we also review the applications of neutralising antibodies in the clinical setting—from predictors of disease severity to serological testing to vaccinations, and finally in therapeutics such as convalescent plasma infusion.

## Background

The Coronavirus disease 2019 (COVID-19) is a disease caused by the etiological agent Severe Acute Respiratory Syndrome Coronavirus 2 (SARS-CoV-2), a newly identified β-coronavirus [1]. SARS-CoV-2 is closely related to SARS-CoV, the coronavirus responsible for the severe acute respiratory syndrome (SARS) epidemic that emerged from 2002 to 2003. SARS-CoV-2 belongs to the lineage B of the *Betacoronavirus* genus in the *Coronaviridae* family [2]. As of 23 August 2021, a total of 211,373,303 COVID-19 cases have been confirmed worldwide, resulting in 4,424,341 deaths [3]. As of 23 August 2021, a total of 4,615,260,567 vaccine doses have been administered [3].

There are four genera in the *Coronaviridae* family, namely α, β, γ, δ [4]. There are seven known coronaviruses that infect humans. HCoV-229E and HCoV-NL63 belong to genus α, while HCoV-OC43, HCoV-HKU1, SARS-CoV, MERS-CoV, and SARS-CoV-2 belong to genus β [5]. Infections with HCoV-229E, HCoV-NL63, HCoV-OC43, and HCoV-HKU1 mainly cause mild respiratory diseases, whereas infections by SARS-CoV, MERS-CoV and SARS-CoV-2 may potentially lead to severe pneumonia and even death [5].

Complete genome sequence homology comparison was used to analyse SARS-CoV-2 samples against several viruses circulating in animals being suspected as likely progenitors of SARS-CoV-2. The SARS-CoV-2 samples shared 96.2% sequence identity with bat-coronavirus (bat-nCoV) RaTG13 [6]. Another bat-nCoV (denoted RmYN02) also shared 93.3% sequence identity with SARS-CoV-2 at the whole genome level [6]. Bats are regarded as the natural reservoir of SARS-CoV-2 due to their biological characteristics as well as the high sequence identity between bat-nCoV and SARS-CoV-2 [6, 7]. However, the intermediate host from which SARS-CoV-2 acquired part of or all the mutations necessary for effective transmission in humans is unknown. There are differences in the genetic sequences encoding the SARS-CoV-2 spike (S) protein that mediates virus entry into human cells, which may account for many of the unique pathogenic properties of SARS-CoV-2 [8, 9]. It has been highlighted in a study that there is a wide phenotypic variation in human antibody responses against SARS-CoV-2 [10], which is important as one needs a standardized and scalable assay for universal and large cohort assessments. To obviate the need to use live virus within a biosafety level 3 (BSL3) facility, an HIV-based lentiviral vector pseudotyped with the SARS-CoV-2 spike protein has been established as a surrogate for use in anti-S neutralising antibody assays in a BSL2 laboratory [10].

SARS-CoV-2 shares some similarities to the two known coronavirus predecessors that caused severe infections in humans to date, i.e. SARS-CoV and MERS-CoV with 79.5% and 50% sequence identity, respectively [9]. However, the SARS-CoV-2 spike protein displays 10 to 20 times greater affinity for angiotensin-converting enzyme 2 (ACE2) receptors on human target cells [9]. The importance of these differences arises from SARS-CoV-2’s infection pathway.

## Immune responses against SARS-CoV-2

The outer surface of SARS-CoV-2 contains the spike (S), matrix (M), and envelope (E) proteins. The S protein plays a role in viral host range and infectivity—it is a critical target for inducing antibodies, particularly neutralising antibodies (NAbs) specific against SARS-CoV-2 [11]. The M protein is the most abundant protein on the viral surface, and is involved in viral budding from the host cell membrane. The E protein is the smallest protein, and is thought to play a role in viral intracellular trafficking and protein assembly [12]. The viral core contains the nucleocapsid protein (NP)—given that NP is “shielded” by viral or cellular membranes, NP antibodies are less likely to directly neutralise SARS-CoV-2 [13].

Like other coronaviruses, the S protein, a large transmembrane homotrimer of approximately 140 kDa on the viral surface, plays an important role in receptor binding and virus entry. The S protein is a class I fusion protein, with each S protomer consisting of S1 and S2 domains. The receptor-binding domain (RBD) is located within the S1 domain [14], and it allows the virus to dock to its cellular receptor, ACE2 [13]. Antibodies that target distinct areas of the S protein inhibit SARS-CoV-2 infection in different ways [11].

The spike S1 subunit mediates viral entry into host cells by binding onto ACE2 [15]. It then fuses with viral and host membranes via the S2 subunit [15]. The binding of S protein to its receptor allows genomic RNA to enter the cytoplasm [16]. Other receptors for SARS-CoV-2, such as CD147 have also been reported [17]. Toll-like receptors (TLRs) are a class of proteins known as pattern recognition receptors that play pertinent roles in the initiation of the innate immune response. They recognise pathogen-associated molecular patterns and represent the first line of defence against infections. TLR-4 recognises the S protein on SARS-CoV-2 and induces the production of pro-inflammatory cytokines through the MyD88-dependent signalling pathway. It is likely that early T cell responses against SARS-CoV-2 may be protective. However, a robust initial response is difficult to elicit because of the efficient innate immune evasion mechanism of SARS-CoV-2 in humans [18]. T cells and inflammatory cytokines may contribute to viral clearance [19], resulting in the more rapid increase in functional lymphocyte counts and higher frequency of CXCR3+ T follicular helper (Tfh) cells, especially in convalescent individuals with more severe disease.

Antibody evolution occurs in germinal centres, where antigens are stored in the form of immune complexes on the surface of follicular dendritic cells for prolonged periods of time, through somatic mutation and selection. Via adaptive immunity, antibodies identify the SARS-CoV-2 S-protein and specifically target and bind to the RBD of S protein within the S1 sub-domain [20]. This activates the antibody-dependent cell cytotoxicity (ADCC) and complement cascade which eliminates infected cells [21]. This binding confers the antibodies with the potential to neutralise viral entry into cells which is crucial in the protective immune response to SARS-CoV-2 infection [22, 23]. Infections can also easily trigger SARS-CoV-2-specific B and T cell responses [20]. The SARS-CoV-2-specific B cell responses elicited in COVID-19 patients lead to the development of specialised antibody-secreting cells (ASCs). The pathogen-specific antibodies are then secreted in large quantities by these ASCs [22].

## Production of neutralising antibodies

### Types of neutralising antibodies

**Fig. 1:**
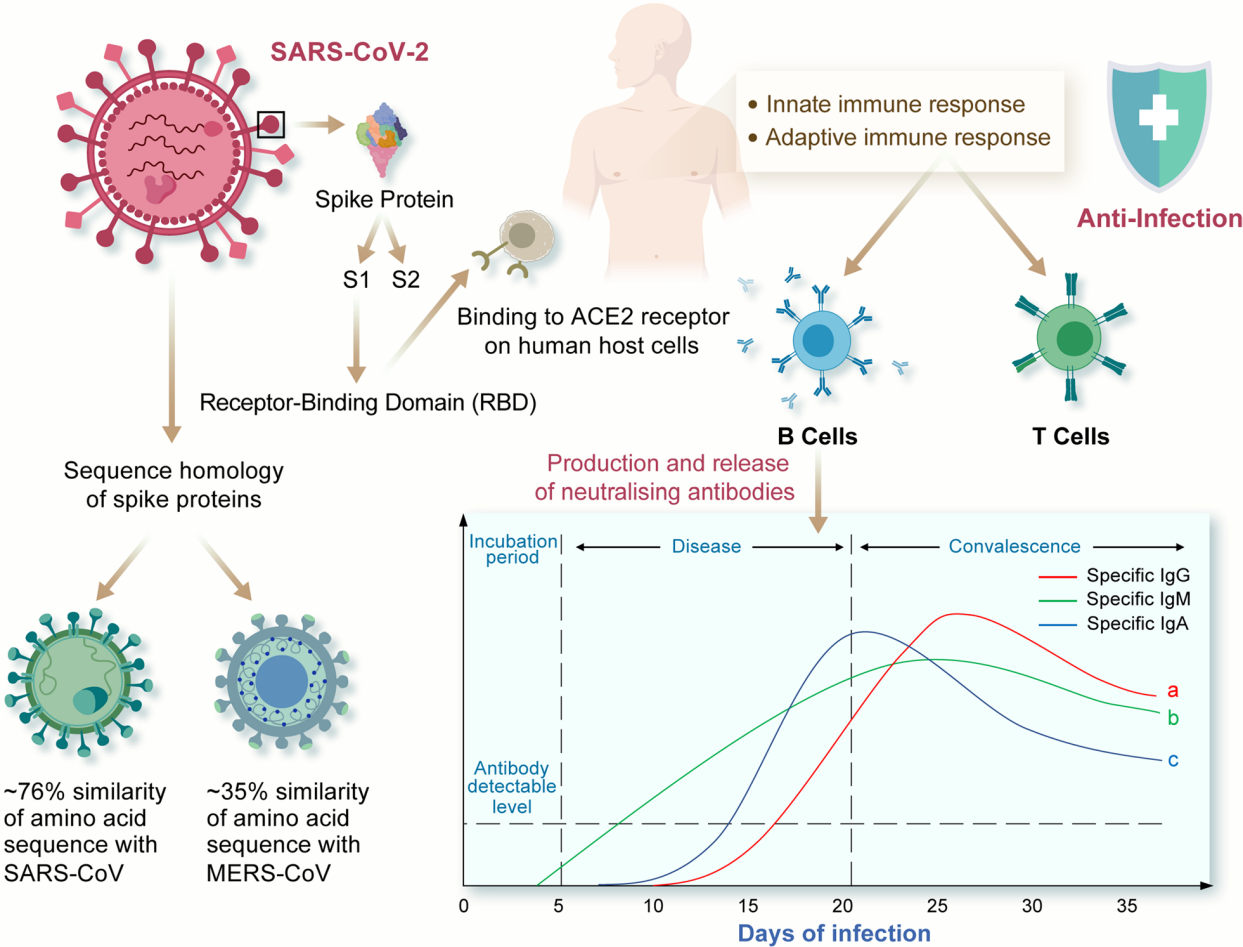
Humoral immune response (IgG, IgM, IgA) profiles of SARS-CoV-2 infections: onset and persistence of neutralising antibodies. **a** Onset within 10–14 days [27–32]; levels remain elevated for weeks [30, 33–38]; peaks at around day 25 [26]. **b** Rises within the first week of infection [30, 31, 33, 39]; lasts 1-2 months before gradually diminishing [15, 30, 38]; peaks at around 20–30 days post-symptom onset [40]. **c** Onset within 6–8 days [39]; lasts 71 days [38]; peaks at around 20–22 days post-symptom onset [39, 40]; high titres in severe COVID-19 cases [32]

Adaptive immunity involves the establishment of immunological memory and the capacity of the immune system to “learn” from many encounters with the same infections—thereby allowing the immune response to become more responsive and effective over time [24]. When all three immunoglobulin classes (i.e. IgG, IgM, and IgA) are found, the maximum neutralisation activity against SARS-CoV-2 is achieved. This is a measure of the ability of the antibodies to work together in a synergistic manner [25] (Fig. 1). Following an infection, anti-SARS-CoV-2 S-specific IgM antibodies are undetectable from days 0–3, and become detectable from day 4 onwards [26]. IgM antibody titre initially rises during the first week of disease onset due to the initial T-dependent humoral response to virus entry and lasts for 20 days to a month before gradually diminishing [15] (Fig. 1). Liu et al. [26] reported that mild cases had a tendency to develop a faster peak of anti-SARS-CoV-2-specific IgM responses at around 17 days, as compared to severe cases whose IgM peaked around 21 days (Fig. 1).

IgG antibody is produced approximately 10–14 days after infection following antigen presentation to T cells and isotype switching [27–32] (Fig. 1). IgG antibody then peaks at around day 25 [26] (Fig. 1), and remains high for weeks [30, 33–38]. IgG has a half-life of only ~ 21 days—hence, sustained antibody titres observed are likely produced by long-lived plasma cells in the bone marrow [13]. From day 15 onwards, there is a statistically significant difference between IgG antibody levels in mild versus severe cases. There is a more robust IgG response against SARS-CoV-2 in severe cases as compared to mild cases [26], and this is correlated with neutralisation levels [27].

Patients with severe COVID-19 show a significant rise in SARS-CoV-2-specific serum IgA and IgG titres after symptom onset. Response of serum IgA against S protein is detectable from 6–8 days after symptom onset [39] and peaks at around 20–22 days post symptom onset [39, 40]. High titres of serum IgA are correlated with severe acute respiratory distress syndrome. On the other hand, patients with mild disease are associated with transient, delayed or even absent production of S-protein-specific serum IgA, suggesting that there is stimulation of mucosal SARS-CoV-2-specific IgA secretion instead of systemic production [32] (Table 1). Serum IgA is the only antibody isotype that rapidly declines in levels, with seropositivity rate decreasing from month 2 onwards [41]. However, neutralising IgA remains detectable in saliva for a longer period, i.e. day 49–73 post-symptoms [42]. IgA is also the main isotype in early neutralising activity of sera [41]. IgA antibodies are dominant in serum, saliva and broncho-alveolar lavage fluid of infected patients, as compared to IgG and IgM. This is also associated with the expansion of IgA plasmablasts with mucosal-homing characteristics [42]. Hence, IgA antibodies are thought to be a major component of NAbs developed in response to SARS-CoV-2 infection [41]. Anti-RBD IgA exhibits a comparable kinetic profile to that of IgG, and its antibody responses are rapid and persistent [40, 43] (Table 1)—perhaps due to the mucosal immune responses in the lungs and intestines. The mucosal IgA production may correlate with viral load, duration of viral exposure, and virus entry route [44, 45].

**Table 1:** Type of antibodies (23 publications)

Study	Study location	Study design	Samples taken (serum/plasma)	Tests conducted	Type of NAbs	Other information
Lagerqvist et al. [46]	Stockholm, Sweden	Case series (*n* = 278)	Serum, plasma	RDTs, IFA, platform-based assays	IgG, IgM	RDTs can detect both IgG and IgM, allowing for the identification of recent and past infections
Goel et al. [47]	Philadelphia, PA, USA	Case series (*n* = 44)	Serum, plasma	ELISA, virus neutralisation assay, flow cytometry and cell sorting, BCR sequencing	IgG	Primary vaccination induced significant increase in IgG levels, further enhanced by the booster dose
Vickers et al. [48]	Iowa City, Iowa, USA	Case series (*n* = 11)	Serum, plasma	CLIA, virus neutralisation assay, ELISA	IgG	10 patients had an average spike-specific IgG level of 4166 AU/mL (range 1235–7854). The non-vaccinated antibody positive subjects screened for this study had an average of 81.7 AU/ml. (*n* = 109)
Yadav et al. [35]	Maharashtra, India	Animal research study	Serum	Clinicoradiological analysis, histopathological examination, immunohistochemistry, virus isolation, ELISA	IgG	IgG levels were detectable from 3rd week post immunisation and were found increasing till the 35th day (7 DPI). Anti-SARS-CoV-2 IgG specific to RBD protein showed a high level of antibody response in the vaccinated group at 28th day post immunisation and 7 DPI
Valdez-Cruz et al. [39]	Mexico City, Mexico	Meta-analysis	Serum	Nil	IgG, IgM, IgA	IgM accumulation is observed within 7 days PSO. IgA titre increases principally between 8 and 21 days PSO. Median time of IgG appearance is 14 days PSO. A significant relationship has been demonstrated between serum titres of anti-S IgA and IgG and the survival of patients in a critical condition
Long et al. [29]	Chongqing, China	Case series (*n* = 285)	Serum, plasma	MCLIA, RT-PCR	IgG, IgM	Within 19 days PSO, 100% of patients tested positive for IgG. Around 20–22 days PSO, the proportion of patients with positive virus-specific IgM peaked at 94.1%. Both IgG and IgM titres plateaued within 6 days following seroconversion
Wang et al. [19]	Chongqing, China	Case series (*n* = 30)	Serum, plasma	Virus neutralisation assays, MCLIA	IgG	SARS-CoV-2–specific NAb titres were low for the first 7–10 days PSO and increased after 2–3 weeks. The median peak time for NAbs was 33 days PSO. NAb titres increased over time in parallel with the rise in IgG levels
Zhao et al. [28]	Shenzhen, China	Case series (*n* = 173)	Plasma	ELISA, IgM-ELISA	IgG, IgM	The seroconversion time of Ab and IgM and IgG Abs appeared consecutively, with a median seroconversion day of 11, 12, and 14, respectively
Garcia-Beltran et al. [49]	Boston, USA	Case series (*n* = 113)	Serum	ELISA, high-throughput SARS-CoV-2 pseudovirus neutralisation assay	IgG, IgM, IgA	Serum IgG Abs appeared almost simultaneously with or sometimes even before serum IgM and IgA Abs PSO. The highest levels of IgG and IgA Abs targeting RBD, and spike were found in severely ill COVID-19 patients who were intubated or had passed away, but there were no significant differences for IgM
Crawford et al. [50]	Seattle, Washington, USA	Case series (*n* = 32)	Serum, plasma	RT-qPCR, virus neutralisation assay, ELISA	IgG, IgM, IgA	At 30 days PSO, hospitalised patients with severe illness exhibited stronger IgG, IgA, and IgM binding reactions than asymptomatic or symptomatic non-hospitalised patients
Margherita Bruni et al. [51]	Milan, Italy	Case series (*n* = 16)	Serum	ELISA, multiplexing analysis of sera cytokines	IgG, IgM	All COVID-19 positive subjects tested positive for the presence of IgG Abs. A few subjects were IgM negative or with an antibody concentration close to the detection limit of the Spike and RBD assay, as compared to the N protein
Seow et al. [40]	London, United Kingdom	Case series (*n* = 65)	Serum	ELISA	IgG, IgM, IgA	51.6% of subjects showed synchronous seroconversion to IgG, IgM, and IgA, whereas some individuals showed singular seroconversion to IgG (9.7%), IgM (9.7%) and IgA (9.7%)
Zhang et al. [31]	Wuhan, China	Case series (*n* = 39)	Serum	RT-qPCR, ELISA	IgG, IgM	Both IgM and IgG titres were relatively low or undetectable on day 0. On day 5, IgM positive rate increased from 50 to 81%, whereas IgG positive rate increased from 81 to 100%
Iyer et al. [38]	Boston, USA	Case series (*n* = 343)	Serum, plasma	ELISA	IgG, IgM, IgA	From days 5 to 14, there was a sharp rise in RBD-specific Abs of all isotypes. IgG levels continued to rise until day 25 after PSO. IgA and IgM responses peaked less than a week earlier than IgG and then declined toward concentrations measured in pre-pandemic samples
Figueiredo-Campos et al. [27]	Lisbon, Portugal	Cohort study (*n* = 2998)	Serum, plasma	ELISA, PCR	IgG	73% of subjects who did not have an IgG response within the first week showed a robust response seven days later (days 9–14). The remaining 27% of subjects still did not show an IgG response in the second week after PSO
Marklund et al. [34]	Gothenburg, Sweden	Cohort study (*n* = 47)	Serum	ELISA, CLIA, flow cytometry, SARS-CoV-2 neutralising antibody assay	IgG	There were significantly higher concentrations of IgG Abs in patients with severe symptoms (mean 107 AU/ml) than in subjects with mild symptoms (mean 65 AU/ml) within 35 days PSO
Cervia et al. [32]	Zurich, Switzerland	Cohort study (*n* = 173)	Serum	ELISA, RT-qPCR, SARS-CoV-2 microneutralisation assay	IgG, IgM, IgA	The mean periods between reported symptom onset and serum collection were 13.5 days in the group of subjects with mild COVID-19 and 20.2 days in the group with severe COVID-19
Yang et al. [52]	Shenzhen, China	Case series (*n* = 479)	Serum	RT-qPCR, ELISA	IgG, IgM, IgA	IgM, IgG, IgA, total antibody, and NAb seropositivity rates at first post-discharge sampling (median: 24 days post-discharge) in recurrent-positive subjects were 37%, 99%, 62%, 99%, and 88% respectively
Wang et al. [30]	Guangzhou/Yangjiang/Qingyuan, China	Case series (*n* = 23)	Plasma	PCR, ELISA, pseudotype-based neutralisation assay, FRNT	IgG, IgM	IgM responses in subjects with severe disease increased within 1–2 weeks PSO and gradually decreased after 4 weeks whereas IgM responses were much lower in mildly ill patients. IgG responses emerged at 10–15 days PSO. Most patients showed high levels of IgG Abs that were maintained for at least 6 weeks
Dispinseri et al. [53]	Milan, Italy	Case series (*n* = 162)	Serum	Flow cytometry, virus neutralisation assay, titration assay, infectivity assay, LIPS assay	IgG, IgM, IgA	IgM and IgA binding to the spike proteins were more frequently detected than IgG during the first two weeks PSO. IgM and IgA levels then progressively declined during follow-up
Marot et al. [41]	Paris, France	Case series (*n* = 26)	Serum	CLIA, ELISA, virus neutralisation assay	IgG, IgM	92.3% of subjects had detectable anti-RBD IgG antibodies and 80.8% had detectable anti-S IgG antibodies. Only 42.3% had detectable anti-N/anti-S IgM antibodies
Wu et al. [14]	Wuhan, China	Case series (*n* = 349)	Serum	RT-PCR, CLIA, virus neutralisation assay	IgM	The positive rate for IgM-S reached a peak of 95% at week 5 and then rapidly decreased to 0% at week 13 fluctuating below 35% thereafter. IgM-N could be detected in 72% of subjects at week 3
Zhou et al. [33]	Hefei, Anhui, China	Case series (*n* = 165)	Serum	ELISA	IgM	SP–IgM showed a tendency to increase from days 1–28, and was detectable on the first day, with a positive rate of 46% and an OD value of 0.3128 ± 0.2365 from days 1–3

*BCR Sequencing* B cell receptor sequencing, *C-ELISA* Competitive enzyme-linked immunosorbent assay, *CLIA* chemiluminescent immunoassay, *COVID-19* Coronavirus disease 2019, *DPI* days post infection, *ELISA* enzyme-linked immunosorbent assay, *FRNT* focus reduction neutralisation test, *IFA* immunofluorescence assay, *IgM-ELISA* IgM μ-chain capture method, *LIPS assay* fluid-phase luciferase immune precipitation assay, *M-CSF* macrophage colony-stimulating factor, *MCLIA* magnetic chemiluminescence immunoassay, *OD* optical density, *PSO* post symptom onset, *RBD* Receptor-binding domain, *RDTs* rapid diagnostic tests, *RT-PCR Test* reverse transcription polymerase chain reaction test*, RT-qPCR* reverse-transcription quantitative polymerase chain reaction, *SARS-CoV-2* severe respiratory syndrome coronavirus 2, *SCF* stem cell factor, *TRAIL* TNF-related apoptosis-inducing ligand

The dynamics of humoral immune response determine the speed of viral elimination. Faster viral clearance is associated with earlier antibody responses—where low initial SARS-CoV-2 RNA was detected in patients who did not have S IgG—suggesting that induction of adaptive humoral response may be dependent on the strength of viral replication [54]. Interestingly, men have higher antibody titres compared to women in the acute phase [27].

Overall, the distribution and variation of IgM, IgG and IgA antibody dynamics may be associated with the patients’ age, gender, co-morbidities, viral load, and other factors that influence disease severity.

### Roles of neutralising antibodies

NAbs are crucial for virus clearance and to achieve protection against SARS-CoV-2 [23]. They may achieve this in several ways—including interfering with virion binding to receptors, blocking virus uptake into host cells, and preventing uncoating of viral genomes in endosome or causing aggregation of virus particles. In the case of COVID-19, however, their roles remain less defined, e.g. the predictive value of neutralisation with regard to disease outcome [40, 55].

Dispensiri et al. [53] concluded that the level of NAbs is correlated to survival and virus control in infected patients. The absence of NAb response early after disease onset showed the strongest correlation with mortality and delayed viral control—more so than the difference in NAb titre. Almost all patients develop NAb by week 4 of infection, and severely ill patients exhibit higher peak, faster and stronger NAb titres compared to mild cases [40, 55, 56]. Hospitalised patients harbour greater NAb titres than mildly symptomatic and asymptomatic patients whose titres were below the detection limit in half of the cases [57].

Early studies showed that most convalescent plasma samples from recovered individuals do not have high levels of neutralising activity. However, convalescent individuals have rare but recurring anti-SARS-CoV-2 RBD antibodies with potent antiviral activity. Notably, a set of RBD-binding monoclonal antibodies (mAb) was derived from convalescent individuals who recovered from COVID-19. These mAb included C121, C144 and C135 which are potent neutralising antibodies against SARS-CoV-2, with half-maximal inhibitory concentrations (IC50) of less than 5 ng/ml [58]. This may be attributed to the possible conformational differences of neutralising epitopes. N-terminal domain (NTD) and RBD are both found on S1 protein. Hence, when NTD-targeting mAb or their fragments target and bind NTD to form a mAb-NTD complex, they avert conformational changes in the viral S protein, thereby blocking membrane fusion and viral entry. Similarly, when RBD-targeting mAbs and nanobodies (Nbs) target and bind to RBD, they form RBD-mAb or RBD-Nb complexes that inhibit the binding of RBD to ACE2. Generally, antibodies which target viral RBD of S protein as their binding site are more potent than those targeting other regions [11]. Amongst various other antibodies studied, human mAb 47D11 was shown to target the conserved core structure of the S1 RBD and to exhibit some cross-neutralising activity through a yet unknown mechanism; and mAb S309 exhibited neutralisation of SARS-CoV-2 through binding to a protein/glycan epitope on SARS-CoV-2 RBD that is distinct from the receptor-binding motif [59, 60]. Hence, there may be broad cross-neutralising epitopes that exist within the lineage B [1]. Other potent neutralising antibodies that bind to ACE2 include P2C-1F11, P2B-2F6 and P2C-1A3, which were most competitive with ACE2 [61].

The correlation between anti-RBD antibody levels and NAbs remains unclear as there are contradictory reports on their association. Billon-Denis et al. [62] studied two patients—one who presented with a strong anti-RBD IgG immune response that correlated with a low and rapidly waning NAb titre, whereas the other had strong IgG anti-RBD immune response, but high NAb titres. Hence, they propose that other host factors (e.g. age, gender, clinical severity) may be more dominant drivers of the immune response as opposed to NAb titres. In contrast, Ju et al. [61] analysed the RBD-specific mAbs of 8 infected patients, and concluded that NAb competing with ACE2 may be a better predictor for virus-neutralising antibody potency rather than binding affinity. Hence, blocking the interaction between RBD and ACE2 may be a useful surrogate for neutralisation. The hindrance of the crystal structure of RBD-bound antibody inhibits viral binding to ACE2, thus blocking viral entry—suggesting that anti-RBD antibodies are mainly viral species-specific inhibitors. Another study also noted the correlation of NAb titres to anti-RBD IgG levels [51].

With regards to seroconversion, patients who did not seroconvert or had reduced or delayed seroconversion had the lowest viral loads [54] or were asymptomatic [27] (Table 1). Long et al. [29] reported that seroconversion for IgG and IgM occurred concurrently or sequentially, and that the titres for both reached a plateau within 6 days after seroconversion. Iyer et al. [38] noted that the median time to seroconversion from symptom onset was nearly 12 days across all three isotypes tested: 10.7 days for IgG (95% CI 9.6–11.9), 11.7 days (95% CI 10.4–13.0) for IgA and 11.9 days (95% CI 10.5–13.4) for IgM. However, IgA and IgM antibodies against RBD were short-lived, with seroreversion of 71 and 49 days after symptom onset. On the other hand, anti-RBD IgG decayed more slowly through 90 days. In hospitalised patients, the median time to seroconversion was faster by 4 days compared with non-hospitalised patients [38]. This is congruent with other observations that seroconversion in mild COVID-19 may take a longer time to mount [13].

Critically ill COVID-19 patients have the highest levels of anti-RBD and anti-spike antibodies. This may be attributed to the host response that includes hyperinflammation and/or uncontrolled viral replication, culminating in an overproduction of antibodies which act as severity biomarkers [49] (Table 1). Patients with severe COVID-19 show significant rise in SARS-CoV-2-specific serum IgA and IgG titres 3 to 5 days after symptom onset [32, 63]. Anti-RBD IgG antibodies are strongly correlated to anti-S neutralising antibody titres (as determined by microneutralisation assays and virus culture), showing that RBD-targeted antibodies can be used to accurately classify individuals with recent versus old infections. This finding concurs with the accumulating body of data [64–67] suggesting that there is development of robust systemic immune memory in individuals with severe infection. Proinflammatory cytokines and antibody titres against RBD and spike protein decreased within a month, but not for nucleocapsid (N) protein [51]. Of note, out of the two major immunogenic proteins, more N proteins are generated, which may account for the earlier appearance of anti-N IgG compared to anti-spike IgG [68]. In particular, larger S1 or RBD relative to N IgG antibody ratios are strongly related with clinically milder illness.

Since there are structural similarities between SARS-CoV-2 and other coronaviruses, several studies explored the possibility of cross-binding and cross-reactivity between them. Some studies [29, 69] reported no cross-reactivity to the S1 sub-unit of SARS-CoV spike antigen in the serum samples of COVID-19 patients. However, COVID-19 patients showed some antibody cross-reactivity to the spike S2 antigens of SARS-CoV and MERS-CoV. Cross-reactivity may be explained in part by the sequence homology of spike proteins, with SARS-CoV-2 sharing ~ 76% of amino acid sequences with SARS-CoV spike and ~ 35% with MERS-CoV spike [69, 70] (Fig. 1). Studies revealed absence of cross-reactivity between SARS-CoV-2 antibodies and the RBDs of SARS-CoV and MERS-CoV, despite their sequence and structural similarities [10, 61]. There was also no observed cross-reactivity of SARS-CoV-2 RBD-targeted antibodies with other circulating coronaviruses such as HKU1, 229E, OC42, NL63 [38]. This suggests that the different RBDs are immunologically distinct [61]. Zhang et al. [69] noted that the serum antibodies from convalescent COVID-19 patients had cross-reactivity with SARS-CoV S1 (38.8%) and SARS-CoV S2 (89.6%). Another study showed no antibody cross-reactivity to SARS-CoV S1 antigen but observed cross-reactivity to SARS-CoV nucleocapsid antigens [29].

## Onset and persistence of neutralising antibodies in SARS-CoV-2 infection

NAbs to SARS-CoV-2 develop in most individuals following infection, but decay over time, and this antibody decay after acute viral antigenic exposure is approximately exponential [71]. Studies have been conducted to assess the humoral immunity to SARS-CoV-2. Some conclude that there is sustained humoral immunity in recovered patients suggesting prolonged immunity, whilst others raise concerns that humoral immunity to SARS-CoV-2 may be short-lived in patients with the moderate disease who constitute the majority of COVID-19 cases [19, 72–74]. There are contradictory reports regarding severity of disease and antibody titre—Zhao et al. [28] (Table 1) reported a correlation, whereas To et al. [63] claimed otherwise.

Following infection or immunization, the initial peak and early decrease in antibodies are common, as most short-lived antibody-secreting plasmablasts responsible for early antibody peak would have died by month 3. Long-lived plasma cells responsible for longer term persistence of antigen-specific antibodies are primarily responsible for antibody production during month 6 and thereafter [75].

In germinal centres, persistent antibody development occurs when B cells are exposed to antigen trapped in the form of immune complexes on follicular dendritic cells. Since follicular dendritic cells do not internalize immune complexes, this type of antigen can persist for a long time. Moreover, low levels of persistent viral antigen may aid antibody development. The persistence of anti-RBD IgA antibodies and continuing antibody development are compatible with the detection of SARS-CoV-2 RNA and protein in the small intestinal epithelium in many infected individuals months after infection [71]. As a result, memory responses are responsible for protection against reinfection and are critical for effective vaccination. In a study to determine if there was altered breadth in antibodies expressed by memory B cell (MBCs), Gaebler et al. [71] compared MBCs at 6.2 months to earlier clonal relatives in binding assays using control and mutant RBDs. Results revealed that 83% of tested antibody clonal pairs displayed an overall increased binding to mutant RBDs at the 6.2-month time-point. Hence, the observation that MBC responses do not disappear after 6.2 months, but rather evolve, strongly suggests that individuals infected with SARS-CoV-2 could mount a rapid and effective response to the virus if they are re-exposed to it [71].

### Duration of neutralising antibodies

Decay of NAbs is thought to occur in two phases: a steeper decline before day 70, and a more gradual decline after day 70 [20]. In a study [71], on the humoral memory response of SARS-CoV-2 in patients at 1.3 months and 6.2 months after infection, IgM showed the greatest reduction in anti-RBD reactivity (53%), followed by anti-RBD IgG (32%), anti-RBD IgA (15%), and anti-N IgG (22%). Another study showed that N-specific IgG decays significantly and more rapidly than S-specific IgG [20]. Hence, it was concluded that although plasma neutralising activity decreases significantly between 1.3 and 6.2 months after infection, antibody titres are still detectable in most infected people, and thus there is the persistence of humoral immunity. During the first 6 months following infection, the anti-SARS-CoV-2 MBC response emerges, with an accumulation of immunoglobulin somatic mutations and the production of Abs with increasing neutralising potency and breadth [13, 71]. L’Huillier et al. [76] also evaluated the persistence of humoral immunity for up to 6 months in individuals with mild COVID-19. At 6 months, 36.7% of their participants had anti-RBD values that were at least twice higher than at 1 month, 4.6% had twofold lower values, and anti-RBD antibodies remained stable for 58.7% of participants. NAbs were detectable in 99.5% of participants 6 months after infection, and mean concentrations of anti-RBD antibody increased gradually over time. RBD-ACE2 inhibiting antibody and anti-RBD antibody concentrations showed a strong correlation [76].

During convalescence, S-specific IgG+ MBCs proliferate, contributing to the additive long-term immunological protection against SARS-CoV-2. Antiviral memory B and T cell responses will almost certainly contribute to long-term immunological protection against COVID-19 [20]. The consistent and sustained increase in S-specific IgG+ MBC frequencies over time is consistent with previous reports of SARS-CoV-2 convalescent participants [77]. Aside from IgG+ and IgA+ MBC populations, most memory immune cell subsets show a general decline in serological immunity over time. More significantly, immunological degradation rates most likely stabilise over time, approaching homeostatic maintenance values. For SARS-CoV-2 infections, however, this set point has yet to be determined. Neutralising antibodies are currently the most generally recognised and accepted protective correlation against a wide range of human respiratory infections. However, there is hitherto no evidence of a link between in vitro neutralisation titres and in vivo protection against SARS-CoV-2 [20].

Röltgen et al. [78] noted that waning antibody levels do not necessarily equate to lost immunity. Local mucosal antibody synthesis in the airways may help prevent or hinder SARS-CoV-2 infection following re-exposure. Even if serum antibodies fade to undetectable levels, infection-stimulated memory B and T cells may generate a faster or more effective response in the future. Initial reinfection reports suggest that SARS-CoV-2 behaves similarly to other community coronaviruses, with reinfection causing milder symptoms than the first infection [79, 80]. It was also observed that outpatients with less severe disease had higher ratios of IgG antibodies targeting spike RBD and S1 domains compared to the N antigen, in the first 2 weeks after symptom onset. This suggests that early humoral immune response focused on spike antigen can help constrain viral infection, even when antibody titres are not yet sufficiently high to be detected in blood [78].

It is highly unlikely that serum antibody persistence is the sole determinant of long-term SARS-CoV-2 protection—with an anamnestic recall of stably maintained memory T and B cell populations likely lowering infection or disease. Further research is needed to determine the quantity, quality, and protective potential of cellular immune responses to SARS-CoV-2. Even mild to severe COVID-19 infections induce substantial cellular immunological memory, as evidenced by a consistent rise in S-specific IgG+ memory B cells reaching a median level of 0.8% of all IgG+ memory B cells after 4 months. Cellular immunological memory is highly likely to minimise the rate of reinfection. More detailed research is needed to better understand how epitope immunodominance changes over time during convalescence [20].

### Neutralising antibodies in reinfection

The phenomenon of reinfection of SARS-CoV-2 presents a case against the protective nature of humoral immunity against this pathogen [81]. The new cases of reinfections suggest that immunity against SARS-CoV-2 may only be temporary and incomplete, given that newly emerging viral variants are able to escape natural immunity [82]. Recovered asymptomatic patients susceptible to reinfection may act as SARS-CoV-2 reservoirs for continuous viral spread [81].

Many factors need to be considered in assessing the effectiveness of an individual’s immune response when reinfected with SARS-CoV-2. Bartsch et al. [83] found that neutralisation, Fc function, and SARS-CoV-2 specific T cell responses are only seen in subjects who elicited RBD-specific antibody titres above a threshold. Only individuals with high IgG titres possess broad and robust RBD-, N- and S-specific humoral immune responses of different subclasses, isotypes, and additional innate immune effector functions. On the other hand, limited humoral immune responses across all three antigens are observed in individuals with low anti-RBD titres. This may be due to the “switch-like” relationship between measured antibody titre and function—a certain level of antibody is needed to generate vigorous humoral and cellular responses. This may be pertinent in conferring individuals with long-lasting protection against SARS-CoV-2 [83].

Another cause of reinfection may be due to the lack of high avidity; avidity being the strength of binding between IgG and its specific target epitope. Avidity is established during affinity maturation, and the failure to achieve high avidity IgG may result in the lack of protective immunity towards infection and disease. For SARS-CoV-2, however, avidity maturation is incomplete, and this is followed by decreased serological response [84]. Due to the high degree of variability in kinetic patterns of IgM and IgG responses towards SARS-CoV-2, acute and past infections cannot be differentiated by only measuring IgM and IgG [85]. Due to the lack of high avidity, cases of reinfection are on the rise, potentially rendering herd immunity difficult to achieve [84]. In terms of correlation between avidity and clinical severity, anti-spike avidity is associated with higher NAb titres. Avidity is significantly higher in hospitalised patients compared to non-hospitalised ones, possibly due to higher viral loads or elevated antibody titres [68]. SARS-CoV-2 RBD IgG avidity was found to be relatively low in most sera collected up to 2 months following symptom onset, with little increase over time [86].

Long et al. [87] proposed that the inference from these results is that memory B cell activation, differentiation, and formation of antibody-secreting B cells (i.e. plasmablasts and plasma cells) may be lacking and not synchronous in recovered individuals. The effective humoral immune response is not conferred to every infected person, and there is no correlation between the magnitude of B cell spot number and virus-specific IgG in peripheral blood. This was evident in their study where positive results were found in only 2 of 13 participants who recovered from asymptomatic infection, and in 6 of 20 who recovered from symptomatic infection.

### Predictors of disease severity

There are several antibody-related predictors of disease severity and/or mortality. Kutsuna et al. [88] reported greater antibody titres associated with male gender, diabetes mellitus, and high maximal levels of C-reactive protein (CRP). Higher CRP levels were correlated to higher antibody titres more so than disease severity. CRP is often used as a sensitive marker of inflammation [88]. In contrast, Gozalbo-Rovira et al. [86] reported weak correlations between antibody assays and inflammatory biomarkers (ferritin, D-dimer, CRP, lactate dehydrogenase (LDH), interleukin-6 (IL-6)). Hence, the latter offers a counter argument against the relationship between the magnitude of antibody response and state of inflammation in COVID-19 patients.

It is believed that the ‘cytokine storm’ plays a key role in disease progression and thus COVID-19 prognosis [89]. Disease severity is also strongly related to NAb levels and anti-S IgG titres [90]. NAb levels in recovered COVID-19 patients are positively linked with the severity of lung injury [91]. The strongest T cell signals and significant neutralising activity are detected in patients with the most severe form of disease—with most patients being old, given that age is a major risk factor [90]. However, Gozalbo-Rovira et al. [86] do not support the association between high SARS-CoV-2 antibody levels and COVID-19 severity. After measuring levels of SARS-CoV-2 RBD IgG and SARS-CoV-2 NAb within the first 30 days after symptom onset, there were no differences between ICU versus non-ICU patients [86].

Batra et al. [12] suggested that IgG antibodies against the N protein are linked to the antibody-dependent enhancement (ADE) phenomenon and increased viremia levels. COVID-19 patients who have recovered may be reinfected, and ADE may play a role during the course of COVID-19 pneumonia [91]. Higher anti-N IgG levels are linked to poorer outcomes such as longer hospital stays, increased chance of ICU admission, longer ICU stays, and increased mortality during hospitalization. COVID-19 patients hospitalized for hypoxemia are likely to have high levels of IgG against the SARS-CoV-2 N protein. Hence, it is deduced that IgG against N protein stimulates a stronger inflammatory response during infection, and may thus be a potential marker for severity [12].

Other possible markers that may be used are the pattern of viral shedding or antibody avidity. There are different patterns of viral shedding and antibody responses in various tissues. In particular, viral shedding is more common in respiratory and faecal material, as opposed to urine and blood. To assess disease severity, antibody responses in urine and other body fluids may be used as markers [30].

### Neutralising antibodies in COVID-19 vaccination

Vaccines need to stimulate the production of antibodies that inhibit the entry of SARS-CoV-2 into cells by blocking either the ACE2-RBD binding interactions or S-mediated membrane fusion [92]. Studies on interactions of SARS-CoV-2 with the host cell and on immune responses after infection identified the S protein as the antigenic target for the development of most vaccines [93]. The development of long-lived memory B cells capable of engendering recall responses is also pertinent if antibodies in circulation fail to provide protection against future exposure [47] (Table 1).

The theoretical risk of aggravating COVID-19 severity via ADE is one possible stumbling block for antibody-based vaccinations and therapeutics. However, no definitive evidence for ADE has been established so far. This concern is raised due to the association of higher antibody titres with more severe clinical disease. ADE works via two distinct pathways that can occur when non-neutralising or sub-neutralising antibodies bind to viral antigens without blocking or eliminating the infection. First, by enhanced antibody-mediated virus uptake into phagocytic cells that express Fc gamma receptor IIa, leading to increased viral infection and replication. Second, by excessive Fc-mediated effector functions or immune complex formation causing exaggerated inflammation and immunopathology—via the secretion of pro-inflammatory cytokines, immune cell recruitment, and complement pathway activation [92]. For COVID-19, the ADE mechanism is likely to involve immune complex formation, complement deposition, and local immune activation. The overactivation of the complement cascade has been shown to contribute to inflammatory lung injury [92].

Goel et al. [47] measured the circulating antibody responses of SARS-CoV-2 naive individuals, pre-vaccination, post-primary vaccination, and post-booster vaccination. They found that in SARS-CoV-2 naive subjects, levels of IgG antibodies specific for full-length spike protein, RBD, or spike-specific memory B cells were undetectable at baseline—however, these increased significantly with primary vaccination and were further enhanced with booster dose. On the other hand, SARS-CoV-2 recovered individuals had detectable levels of anti-spike and anti-RBD IgG at baseline, and these antibody and memory B cell responses increased significantly after the first vaccine dose, but there was no increase in circulating antibodies, neutralising titres or antigen-specific memory B cells after the second dose. Remarkably, levels of anti-RBD IgG in SARS-CoV-2 naive and SARS-CoV-2 recovered patients were similar, one week after the booster dose. In another study of volunteers who received two doses of the mRNA vaccine against SARS-CoV-2, plasma neutralising activity and relative numbers of RBD-specific memory B cells of vaccinees were equivalent to those who recovered from natural infection. However, neutralising antibody activity against certain viral variants-of-concern was reduced by a small but significant margin [94]. Vickers et al. [48] reported that non-vaccinated subjects had an average spike-specific antibody level of 81.7 AU/ml, whereas their vaccinated counterparts all had antibody levels greater than 1200 AU/ml and at least tenfold higher than before vaccination.

## Serological tests of antibodies

Serological assays for infectious agents have two important and separate applications: firstly, to diagnose chronic infections, and secondly, to determine prior infection or immunisation status which may be used to predict immunity against future infection [95]. Serology tests detect the presence of IgA, IgM and IgG antibodies against SARS-CoV-2, and facilitate profiling of early humoral response in patients [96]. There are three main platforms in which serological tests are coupled with purified proteins of SARS-CoV-2—i.e. lateral flow immunoassay (a point-of-care or POC test), chemiluminescent immunoassay (CLIA), and enzyme-linked immunosorbent assay (ELISA) [96].

ELISA remains the gold standard for antibody detection in view of its high flexibility and sensitivity [27]. Many serological assays employ two structural proteins as target antigens—namely, the nucleoprotein and the spike protein [13]. Specificity and sensitivity were high for anti-RBD IgG and IgA (92–97%), but slightly lower for IgM and for ELISA using spike and N proteins (90–85%) [51]. Antibody determination is influenced by factors such as methods of viral inactivation, the complexity of RBD, monomeric, and dimeric mixtures. Higher levels of expression are attained with the comparatively smaller RBD as opposed to the spike protein—thereby rendering RBD the preferred choice to study [27].

Studies have investigated the differences between the rapid COVID-19 test kit versus the CLIA quantitative antibody test. The results differed greatly—8.8% of subjects tested positive using the rapid test kit (with 92% sensitivity, 97% specificity) compared with 0.9% using the CLIA quantitative IgG antibody test. Hence, a protocol should be adopted when rapid test kits are deployed in hospitals and communities—i.e. a standard follow-up for subjects who test IgM positive which currently is to perform real-time reverse transcriptase polymerase chain reaction (RT–PCR) testing. It is vital to evaluate sensitivity of subclinical infections with sera from asymptomatic RT-PCR-positive individuals as positive control, and to assess specificity with sera collected before COVID-19 as negative control [97]. A study demonstrated that anti-SARS-CoV-2 S-RBD IgG CLIA has outstanding linearity for range of values within and above the cut-off points, rendering it especially useful for vaccinated individuals where antibody values are above the detection limit [98]. Another study comparing the performance of GFP-reporter-based pseudotyped virus neutralisation assay versus four commercial immunoassays targeting SARS-CoV-2 S protein yielded results of 100% specificity for COVID-19 diagnosis, and correlation between neutralising antibody titres and SARS-CoV-2 IgG levels [99].

There are certain limitations with respect to the utility of serological testing. Individual disparities in antibody titres may be influenced by differences in antigen exposure. Relationships between viral load and antibody responses are difficult to establish due to a variety of factors. These factors include variations in viral load trajectories; time of diagnosis and sampling relative to infection; sampling efficiency using swab samples; and the relationship between nasal viral load and systemic antigen exposure. As a result, serological data cannot always be harnessed to precisely predict the trajectory of neutralising antibody levels [95]. Antibodies that arise during the infection may be difficult to detect in the early stages, but they persist long after infection has passed. Hence, assays that measure these antibodies should provide additional information on the fraction of individuals who have been infected. Due to the time taken for adequate antibody response to develop, false negative results may occur depending upon when sampling was done [100].

Whilst serological tests are not critical in the early diagnosis of infection, and cannot assume the major role of direct viral testing to diagnose an acute infection [101], they are crucial in providing data on pathogen exposure, prevalence of infection, and selection of convalescent plasma donors to serve a therapeutic function [98]. By measuring SARS-CoV-2 IgG levels using immunoassays targeting the S protein in sera from infected patients, the degree of correlation between neutralising antibody binding the SARS-CoV-2 S protein and producing the most potent antibodies for virus neutralisation may be estimated [102]. Serological tests are useful for acute diagnosis of COVID-19 infection in patients who present late, or when the sensitivity of RT-PCR testing is decreasing [101].

## Transfer of antibodies using convalescent sera

In efforts to treat severely ill COVID-19 patients, passive transfer of antibodies from convalescent COVID-19 patients has been employed. A study [103] on 6 convalescent donors suggested that recovered COVID-19 patients may serve as suitable donors for convalescent plasma (CP) therapy, provided they fulfil other blood donation criteria. All the 6 participants showed positive IgM results. However, IgM as a serological marker to represent recent or current infection may not be suited as part of the mandatory criteria for CP donation. Currently, there is insufficient information on the relative neutralising capacity of antibodies from convalescent donors, thus affecting standardisation in the implementation of CP therapy.

Benefits may be reaped from using CP treatment. Administering CP in older adult patients within 72 h of symptomatic COVID-19 reduces the risk of progression from early or mild stage to severe respiratory disease by 48%. There is also a dose-dependent IgG effect in CP infusion, and early infusion may bridge the time gap between recovery and vaccination [104]. In a clinical trial where severely ill COVID-19 patients were transfused with plasma, the variability in clinical response and recipient antibody titres post-transfusion suggests that CP therapeutic efficacy is dependent on when treatment is administered, and the composition of CP [105].

In recruiting CP donors, the factors for high viral neutralisation to consider include older age, male gender, and patients with more severe infection and higher CRP levels [57, 106, 107]. To ensure a high likelihood of achieving sufficiently high RBD-specific IgG titres, Li et al. [108] also recommend the following selection criteria for optimal CP donation, i.e. 28 days after symptom onset with fever more than 3 days or temperature over 38.5℃. This therapeutic method may yield better results than anti-spike monoclonal antibodies due to the rising number of variants. However, it is costly and requires more extensive equipment and personnel [107]. Wang et al. [109] identified RBD-targeting antibodies from convalescent donors with potent neutralising activity against 23 variants of SARS-CoV-2. Antibody avidity represents another potential screening parameter to identify CP donors. This is based on data that anti-spike IgG avidity has a stronger association with neutralising titres—although more research is needed to justify this factor [68].

## Limitations of this review

Several limitations must be considered when reviewing information presented in this review. The information presented here is based on reports published before May 2021. Due to the evolving nature of COVID-19, coupled with a multitude of study techniques, statistical approaches, demographic characteristics and geographical locations, interpretation of certain data may have been confounded. Another limitation is that the contribution and cooperation of cell-mediated immunity with neutralising antibodies were not considered. For example, CD8+ memory T cells that specifically recognize conserved epitopes from previous seasonal coronavirus infections correlate with milder COVID-19 [110]. Nevertheless, as the pandemic continues to evolve, it is pertinent to better understand antibodies and their functions, as they play critical roles—from infection, to persistence, to reinfection, and finally to therapeutic applications.

## Conclusion

In order to better understand the pathophysiology of COVID-19, it is vital to understand the immune responses against SARS-CoV-2 and how different antibodies are generated during infection. Information on the onset, peak and persistence of various antibodies is useful in evaluating host immunity against SARS-CoV-2.

More importantly, the neutralising antibodies and their persistence are critical in determining clinical severity, including how they influence chances of reinfection in recovered individuals. Moving forward, it is vital that these data are harnessed to improve therapeutic efforts such as using convalescent sera from recovered patients. Given that the understanding of SARS-CoV-2 is constantly evolving and dynamic, with multiple new mutations emerging, it is therefore pertinent to better understand the underlying mechanisms and clinical applications of antibodies.

## Data Availability

Not applicable.

## Abbreviations

Ab: Antibody
ACE2: Angiotensin-converting enzyme 2
ADCC: Antibody-dependent cell cytotoxicity
ADE: Antibody-dependent enhancement
ASCs: Antibody-secreting cells
bat-nCoV: Bat-coronavirus
BSL3: Biosafety level 3
CLIA: Chemiluminescent immunoassay
COVID-19: Coronavirus disease 2019
CP: Convalescent plasma
CRP: C-reactive protein
E protein: Envelope protein
ELISA: Enzyme-linked immunosorbent assay
IC50: Half-maximal inhibitory concentrations
Ig: Immunoglobulin
IL: Interleukin
LDH: Lactate dehydrogenase
M protein: Matrix protein
mAb: Monoclonal antibodies
MBCs: Memory B cells
N protein: Nucleocapsid protein
NAbs: Neutralising antibodies
Nbs: Nanobodies
NP: Nucleoprotein
NTD: N-terminal domain
POC: Point of care
RBD: Receptor-binding domain
RT-PCR: Real-time reverse transcriptase polymerase chain reaction
SARS: Severe acute respiratory syndrome
SARS-CoV-2: Severe respiratory syndrome coronavirus 2
S-protein: Spike protein
Tfh: T follicular helper
TLRs: Toll-like receptors
